# Multi-Robot System for Cooperative Tidying Up with Mobile Manipulators and Transport Agents

**DOI:** 10.3390/s25113269

**Published:** 2025-05-22

**Authors:** Jae-Bong Yi, Shady Nasrat, Dongwoon Song, Joonyoung Kim, Seung-Joon Yi

**Affiliations:** Department of Electrical Engineering, Pusan National University, Busan 46241, Republic of Korea; niteofhunter@pusan.ac.kr (J.-B.Y.); shadyloai@pusan.ac.kr (S.N.); dongwoon@pusan.ac.kr (D.S.); kjykjy98@pusan.ac.kr (J.K.)

**Keywords:** heterogeneous multi-agent pickup and delivery, cooperating robots, multi-robot task allocation

## Abstract

This paper presents a system in which mobile manipulators and transport agents cooperate to solve a multi-agent pickup and delivery (MAPD) problem. The primary objective is to allocate appropriate tasks to heterogeneous robots by considering their capabilities and states. Unlike previous studies that focused on homogeneous teams or assigned distinct roles to heterogeneous robots, this work emphasizes synergy through cooperative task execution. A key feature of the proposed system is that mobile manipulators behave differently depending on whether they are paired with a transport agent. Additionally, rather than generating a full trajectory from start to end, the system plans partial trajectories, allowing dynamic re-pairing of transport agents through an auction algorithm. After re-pairing, new starting nodes are defined, and the following trajectory is updated accordingly. The proposed system is validated through simulations, and its effectiveness is demonstrated by comparing it against a baseline system without dynamic pairing.

## 1. Introduction

In recent years, multi-robot systems are utilized in various fields, such as logistics [1,2], rescue [3,4], medical care [5,6], and agriculture [7,8]. For these domains, which require the coordination of multiple robots, solving MAPD problems is an important part of the process. Current research in MAPD focuses on developing efficient algorithms for task allocation and path planning to optimize efficiency [9,10]. These studies aim to minimize travel distance, task completion time, and resource consumption while ensuring a collision-free trajectory for multiple agents [11,12,13].

However, most existing approaches assume homogeneous robot teams [7,14] or assigning heterogeneous robots to distinct tasks [15,16,17,18]. Although some studies address heterogeneous robots performing the same task, they rarely focus on performing assigned tasks cooperatively [3,5,19].

In this context, we propose a multi-robot system that performs tidying-up tasks by integrating two heterogeneous robot types: mobile manipulators and transport agents. The system is designed to support synergistic collaboration between these robots.

**Figure 1:**
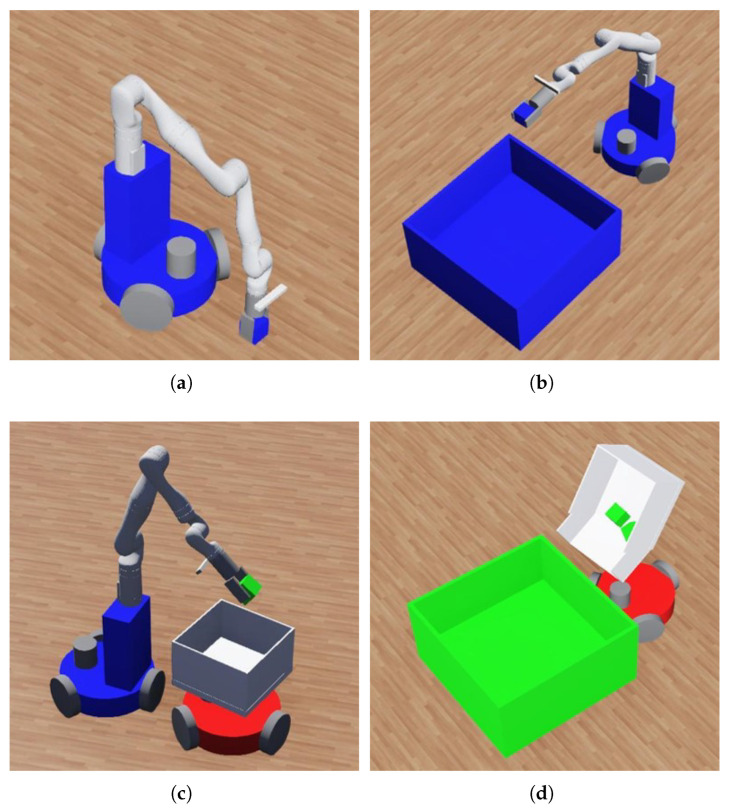
Capabilities of the mobile manipulator (**a**,**b**) and the transport agent (**c**,**d**): (**a**) picking up an object; (**b**) delivering an object at the destination; (**c**) receiving an object from a paired mobile manipulator; (**d**) delivering multiple objects to the destination.

Figure 1 shows the capabilities of mobile manipulators and transport agents. In this system, a mobile manipulator can pick up an object and deliver it to the destination or hand it to a transport agent. In contrast, a transport agent cannot pick up objects themselves but can receive them from manipulators. However, unlike mobile manipulators, they can deliver multiple objects to the destination in a single trip.

Unlike previous approaches that assign independent tasks to each robot type, this system enables pairing these two types to form collaborative units and create complementary capabilities by integrating trajectory optimization and auction-based task allocation.

In generating an optimized trajectory, the objective function consists of traveling and object-handling time without considering the process of finding new partners. The planning conditions are different depending on whether a transport agent is paired (carrying multiple objects) or unpaired (carrying only one object). Additionally, the system generates a partial trajectory corresponding to the number of objects to deliver in a single expectation.

After matching a new pairing partner and generating the sequence trajectory, new starting nodes are defined at the positions where robots are expected to be located after a certain amount of time. From these nodes, a new trajectory is generated.

### 1.1. Related Work

Several works have been proposed to solve the MAPD problem. Authors in [14] present the MAPD approach that robots can run in a peer-to-peer fashion. By distributing computation to multiple robots using primal decomposition [20,21,22], this method enables a reduction in computations without a central node. However, this work assumes the use of homogeneous robots. Additionally, duplicate access to delivery points is not considered in the optimization, and all robots should start their task at the same starting point. Furthermore, they conducted only abbreviated experiments, without pick-and-place, using mobile robots without manipulators. Authors in [23] show a game-theoric multi-robot task allocation framework for multi-robot trash collection in dynamically changing environments. In this work, robots choose their working area based on a decision-making algorithm consisting of a payoff (reward) mechanism, Poisson clock, and task revision protocol. Another MAPD framework introduced in [7] shows a practical example of using a multi-objective discrete artificial bee colony (MODABC) algorithm in orchards. However, both [7,23] favor the use of homogeneous robots in solving MAPD problems.

Meanwhile, other studies deal with multi-robot systems with heterogeneous robots. Authors in [3] show task allocation with heterogenous robots by considering their capabilities and states in a rescue scenario, and they prove its validity by applying it to *Capture the Flag* game. Authors in [5] present task allocation and path planning of heterogeneous robots in medical scenarios. This work introduces Intensive Inter-task Relationship Tree Search (IIRTS) to perform fast task allocation and enable real-time implementation. However, they only present the methods to place heterogeneous robots in appropriate places and do not present how to perform the tasks at the arrival point.

Research in [15,16,17,18] deals with a topic similar to this work that solves the MAPD problem with mobile manipulators and transport agents. Although they solve this problem in various ways (e.g., weighted block sequence graph, constructive heuristics, and recurrent open shop scheduling), these works have limitations in restricting the movement range of mobile manipulators to near storage units. For this reason, these robots only take out items from storage at designated points. Furthermore, as this work assumes that all robots are operated in a structured factory setting with a grid path, applying them in non-structured environments is challenging.

### 1.2. Contributions

In order to implement the MAPD using only optimization, the timing and location of pairings should be considered in the optimization condition. However, as this method requires an extremely high amount of computation, it is impossible to use in practice. To address this issue, we introduce a multi-robot control system that combines optimization and auction algorithms. This work provides the following contributions.

1. We propose a novel MAPD system that enables cooperative task execution between mobile manipulators and transport agents in the same space.
2. The system integrates trajectory optimization and an auction-based task allocation mechanism to adjust the pairing of robots dynamically.
3. Unlike previous full-trajectory approaches, our method uses a horizon-based partial trajectory planning strategy that enables dynamic pairing and re-optimization during execution with reduced computational costs.
4. The system applies different planning conditions to mobile manipulators depending on whether they are currently paired with a transport agent or are operating independently. On the other hand, many previous works assume robots have fixed roles or behave in the same way.
5. The proposed system is validated through realistic simulations, demonstrating the algorithm’s effectiveness.

## 2. System Overview

**Figure 2:**
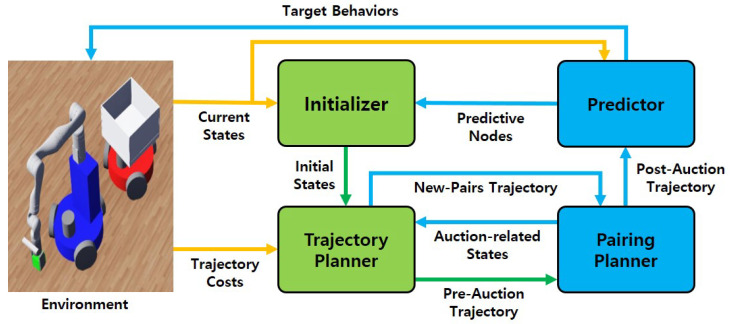
Overview of system structure: yellow arrows, green arrows, and blue arrows present the state flows starting from the environment, initializer, and predictor, respectively.

The overall system structure, shown in Figure 2, is composed of four main components: the initializer, trajectory planner, pairing planner, and predictor.

At the beginning of an operation, the initializer processes the current states of the environment—such as robot positions and object distributions derived from GPS sensors—and derives the initial states necessary for trajectory planning. These are passed to the trajectory planner, which generates a robot’s optimal path up to a predefined planning horizon without considering pairing updates.

Next, the pairing planner uses an auction-based algorithm to determine new pairing partners for mobile manipulators and transport agents. It evaluates pairing options based on cost and availability and generates a trajectory that reflects these new pairings. These are then merged with the initial pre-auction trajectory to form the post-auction trajectory, which is passed to the predictor.

The predictor analyzes the post-auction trajectory and estimates predictive nodes, which are used by the initializer in the next planning cycle. It also contributes to determining the target behaviors of the robots by combining trajectory information with the current environmental context.

Finally, using the combined trajectory and current state information, the system derives the target actions for each robot.

## 3. Pre-Auction Trajectory Planning

We formulate the problem using the notations defined in Table 1 to generate optimal trajectories up to a predefined planning horizon without considering pairing updates.

**Figure 3:**
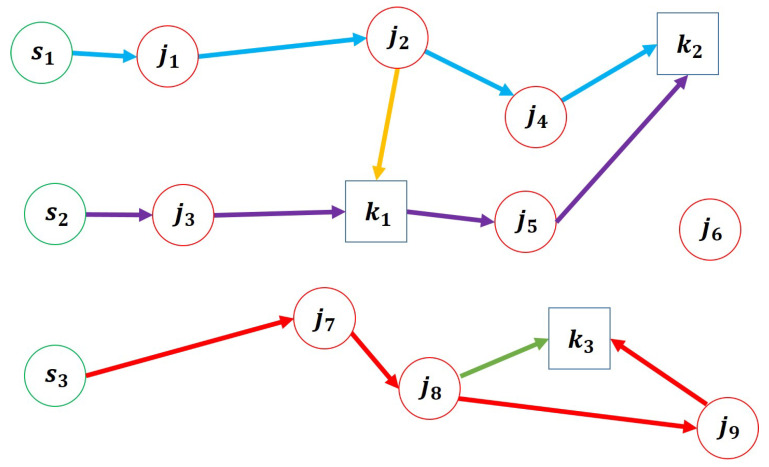
Pre-auction trajectory with H=8: blue arrows, purple arrows, and red arrows represent the trajectory of mobile manipulators $i_{1}$, $i_{2}$, and $i_{3}$, respectively, and yellow and green arrows represent the trajectory of transport agents separated from $i_{1}$ and $i_{3}$, respectively.

An example of such a trajectory is illustrated in Figure 3, which includes three types of nodes: *s* for starting positions, *j* for object locations, and *k* for delivery destinations. In this example, mobile manipulators $i_{1}$ and $i_{3}$, which start at $s_{1}$ and $s_{3}$, are initially paired with transport agents, while $i_{2}$, starting at $s_{2}$, is not.

The destination node $k_{1}$ is assigned to the objects from $j_{1}$ to $j_{3}$, $k_{2}$ to the objects from $j_{4}$ to $j_{6}$, and $k_{3}$ to those from $j_{7}$ to $j_{9}$.

### 3.1. Initializer

Before computing the optimized trajectory, it is necessary to initialize the state of each mobile manipulator *i*. Algorithm 1 outlines this process, where the initial cost $c_{i}^{\mathrm{init}}$, starting node $s_{i}$, paired transport agent $\tau_{i}$, and initially loaded objects $l_{i}^{\mathrm{init}}$ are determined.
**Algorithm 1** Initializer.1:fori∈Ido2:  ifpredictivenodesexistthen3:    $c_{i}^{\mathrm{init}}\leftarrow GETINITIALCOST\left(s_{i}^{\mathrm{pred}},\Pi_{i}^{\mathrm{prev}}\right)$4:    $s_{i}\leftarrow GETLASTNODEINPREDICTION\left(s_{i}^{\mathrm{pred}},\Pi_{i}^{\mathrm{prev}}\right)$5:    $\tau_{i}\leftarrow GETPAIREDTRANSPORTAGENT\left(\tau_{i}^{\mathrm{pred}},\Pi_{i}^{\mathrm{prev}}\right)$6:    $l_{i}^{\mathrm{init}}\leftarrow l_{i}^{\mathrm{pred}}$7:  else8:    $c_{i}^{\mathrm{init}},\;s_{i},\;\tau_{i},\;l_{i}^{\mathrm{init}}\leftarrow 0,\;p_{i}^{\mathrm{now}},\;\tau_{i}^{\mathrm{now}},\;l_{i}^{\mathrm{now}}$9:endfor

If predictive states are available, $c_{i}^{\mathrm{init}}$ is computed based on the predictive node $s_{i}^{\mathrm{pred}}$ in the previous trajectory $\Pi_{i}^{\mathrm{prev}}$; $s_{i}$ is defined as the last accessing node during the prediction period; $\tau_{i}$ is considered paired with *i* if $\tau_{i}^{\mathrm{pred}}$ arrives at the pairing node within the prediction range; and $l_{i}^{\mathrm{init}}$ is set to its predictive value $l_{i}^{\mathrm{pred}}$. (This process is explained in more detail in Section 4.2).

In contrast, if predictive states are unavailable, $c_{i}^{\mathrm{init}}$ is set to zero; $s_{i}$ is assigned to the data received at the current position from an embedded GPS $p_{i}^{\mathrm{now}}$; and $\tau_{i}$ and $l_{i}^{\mathrm{init}}$ are also set to their current states $\tau_{i}^{\mathrm{now}}$ and $l_{i}^{\mathrm{now}}$, respectively.

### 3.2. Trajectory Planner

After determining the initial states, the following objective function and constraints are defined to generate the trajectory.

#### 3.2.1. Objective Function


(1) $$\min_{i\in I,\;j\in J}\left\{w\max_{i\in I}C_{i}+\left(1-w\right)\sum_{i\in I}C_{i}\right\}$$

Equation (1) describes the objective function used to minimize the completion time and the total cost of all robots. These two components can be balanced using the weight parameter *w*. A higher value of *w* emphasizes minimizing the maximum completion time, while a lower value prioritizes the reduction of the total cost.

#### 3.2.2. Traveling Constraints


(2) $$x_{i}^{j_{a}kj_{b}}=0,\;x_{i}^{j_{a}j_{b}}=0,\;x_{i}^{kj_{a}j_{b}}=0,\;\mathrm{where}\;j_{a}=j_{b},\forall i\in I$$

(3) $$x_{i}^{j_{a}kj_{b}}x_{i}^{j_{b}kj_{a}}=0,\;x_{i}^{j_{a}j_{b}}x_{i}^{j_{b}j_{a}}=0,\;x_{i}^{kj_{a}j_{b}}x_{i}^{kj_{b}j_{a}}=0,\;\forall i\in I$$

(4) $$x_{\neg i_{p}}^{j_{a}j_{b}}=0,\;x_{\neg i_{p}}^{kj_{a}j_{b}}=0,\;\forall i_{p}\in I_{p}$$

(5) $$x_{i_{p}}^{j_{a}j_{b}}=0,\;\forall i_{p}\in I_{p},\;\mathrm{where}\;k_{j_{a}}\neq k_{j_{b}}$$

(6) $$x_{i_{p}}^{sj_{a}}x_{i_{p}}^{j_{a}kj_{b}}=0,\;\sum_{\begin{array}{c} j_{a},j_{b}\in J \end{array}}x_{i_{p}}^{kj_{a}j_{b}}\leq 1,\;\forall i_{p}\in I_{p}$$

Equations (2) and (3) show that the mobile manipulators cannot stop at a specific node and cannot return to the previously visited node.

Equations (4) and (5) describe the conditions when a mobile manipulator travels directly between object nodes. Equation (4) describes that performing such direct travels is restricted for the mobile manipulators not paired with the transport agents. However, even if paired, as described in Equation (5), object nodes $j_{a}$ and $j_{b}$ should have the same destination node in performing direct travels without separation.

Equation (6) describes the conditions for the mobile manipulators paired with transport agents: they cannot travel to an object node via a destination node immediately after its first movement. Additionally, separating tasks can only be performed once for the paired mobile manipulators.

#### 3.2.3. Manipulation Constraints


(7) $$\sum_{\begin{array}{c} j\in J \end{array}}x_{i}^{sj}\leq 1,\;\forall i\in I$$

(8) $$\sum_{\begin{array}{c} i\in I \end{array}}x_{i}^{jk}\leq 1,\;\forall j\in J$$

(9) $$x_{i}^{sj}=x_{i}^{sj}\sum_{\begin{array}{c} j^{\prime }\in J \end{array}}x_{i}^{j^{\prime }k},\;\forall i\in I,\forall j\in J$$

(10) $$\left\{\sum_{\begin{array}{c} i\in I \end{array}}x_{i}^{sj_{a}}+\sum_{\begin{array}{c} i\in I \end{array}}\sum_{\begin{array}{c} j_{b}\in J \end{array}}\left(x_{i}^{j_{b}j_{a}}+x_{i}^{kj_{b}j_{a}}+x_{i}^{j_{b}kj_{a}}\right)\right\}\leq 1,\;\forall j_{a}\in J$$

(11) $$\sum_{\begin{array}{c} j_{a}\in J \end{array}}\left\{\sum_{\begin{array}{c} i\in I \end{array}}x_{i}^{sj_{a}}+\sum_{\begin{array}{c} i\in I \end{array}}\sum_{\begin{array}{c} j_{b}\in J \end{array}}\left(x_{i}^{j_{b}j_{a}}+x_{i}^{kj_{b}j_{a}}+x_{i}^{j_{b}kj_{a}}\right)\right\}=H$$

Equations (7)–(9) describe that if a mobile manipulator starts a task, it can only execute one starting task $x_{i}^{sj}$ and one final task $x_{i}^{jk}$. However, these constraints alone may result in multiple robots performing the same task. This issue is addressed by Equation (10), which prevents such overlaps by enforcing exclusivity in task execution. Moreover, Equation (10) is extended to Equation (11) by summing over all object nodes, thereby defining the horizon that the number of objects handled in a single planning. In Equation (11), the horizon is determined based on available computing power.

#### 3.2.4. Connecting Constraints


(12) $$x_{i}^{sj_{a}}=x_{i}^{sj_{a}}\left\{\sum_{\begin{array}{c} j_{b}\in J \end{array}}\left(x_{i}^{kj_{a}j_{b}}+x_{i}^{j_{a}j_{b}}+x_{i}^{j_{a}kj_{b}}\right)+x_{i}^{j_{a}k}\right\},\;\forall j_{a}\in J,\forall i\in I$$

(13) $$x_{i}^{j_{a}k}=x_{i}^{j_{a}k}\left\{\sum_{\begin{array}{c} j_{b}\in J \end{array}}\left(x_{i}^{kj_{b}j_{a}}+x_{i}^{j_{b}j_{a}}+x_{i}^{j_{b}kj_{a}}\right)+x_{i}^{sj_{a}}\right\},\;\forall j_{a}\in J,\forall i\in I$$

(14) $$x_{i}^{j_{a}kj_{b}}=x_{i}^{j_{a}kj_{b}}\left\{x_{i}^{sj_{a}}+\sum_{\begin{array}{c} j_{c}\in J \end{array}}\left(x_{i}^{kj_{c}j_{a}}+x_{i}^{j_{c}kj_{a}}\right)\right\},\;\forall j_{a}\in J,\forall i\in I$$

(15) $$x_{i}^{j_{b}kj_{a}}=x_{i}^{j_{b}kj_{a}}\left\{x_{i}^{j_{a}k}+\sum_{\begin{array}{c} j_{c}\in J \end{array}}x_{i}^{j_{a}kj_{c}}\right\},\;\forall j_{a}\in J,\forall i\in I$$

(16) $$x_{i}^{j_{a}j_{b}}=x_{i}^{j_{a}j_{b}}\left\{x_{i}^{sj_{a}}+\sum_{\begin{array}{c} j_{c}\in J \end{array}}x_{i}^{j_{c}j_{a}}\right\},\;\forall j_{a}\in J,\forall i\in I$$

(17) $$x_{i}^{j_{b}j_{a}}=x_{i}^{j_{b}j_{a}}\left\{x_{i}^{j_{a}k}+\sum_{\begin{array}{c} j_{c}\in J \end{array}}\left(x_{i}^{j_{a}j_{c}}+x_{i}^{kj_{a}j_{c}}\right)\right\},\;\forall j_{a}\in J,\forall i\in I$$

(18) $$x_{i}^{kj_{a}j_{b}}=x_{i}^{kj_{a}j_{b}}\left\{x_{i}^{sj_{a}}+\sum_{\begin{array}{c} j_{c}\in J \end{array}}x_{i}^{j_{c}j_{a}}\right\},\;\forall j_{a}\in J,\forall i\in I$$

(19) $$x_{i}^{kj_{b}j_{a}}=x_{i}^{kj_{b}j_{a}}\left\{x_{i}^{j_{a}k}+\sum_{\begin{array}{c} j_{c}\in J \end{array}}x_{i}^{j_{a}kj_{c}}\right\},\;\forall j_{a}\in J,\forall i\in I$$

Equations (12)–(19) define the connecting constraint that connected edges should share the same node. These constraints enforce the continuity of movement by requiring the connections between nodes to align with respective task assignments. Additionally, Equation (19) specifies that if the mobile manipulators separate from their paired transport agents, they are no longer allowed to move directly between object nodes.

#### 3.2.5. Ordering and Capacity Constraints


(20) $$o_{i}^{j_{b}}=o_{i}^{j_{a}}+1-n\left(J\right)\left(1-x_{i}^{j_{a}j_{b}}-x_{i}^{j_{a}kj_{b}}\right),\;\forall j_{a}\in J,\forall i\in I$$

(21) $$\sum_{\begin{array}{c} j_{a}\in J \end{array}}\sum_{\begin{array}{c} j_{b}\in J \end{array}}x_{i}^{j_{a}j_{b}}<\left(L^{\max}-l_{i}^{\mathrm{init}}\right),\;\forall i\in I$$

Equation (20) defines the ordering constraints to prevent the generation of infinite loops in the trajectory (e.g., $x_{i}^{j_{a}kj_{b}}=1$, $x_{i}^{j_{b}kj_{c}}=1$, and $x_{i}^{j_{c}kj_{a}}=1$), by assigning an execution order using the Miller–Tucker–Zemlin (MTZ) formulation [24].

Additionally, since the number of loaded objects increases by one whenever the robot travels directly between two object locations, the number of such direct travels for *i* is bounded by its maximum carrying capacity and the number of initially loaded objects, as expressed in Equation (21).

#### 3.2.6. Total Cost


(22) $$\begin{array}{c} C_{i}=c_{i}^{\mathrm{init}}+\sum_{j\in J}c_{i}^{sj}x_{i}^{sj}+\sum_{j\in J}c^{jk}x_{i}^{jk}+\sum_{\begin{array}{c} j_{a},j_{b}\in J \end{array}}\left(c^{j_{a}k}+c^{kj_{b}}\right)x_{i}^{j_{a}kj_{b}}\;+\\ \sum_{\begin{array}{c} j_{a},j_{b}\in J \end{array}}c^{j_{a}j_{b}}x_{i}^{j_{a}j_{b}}+\sum_{\begin{array}{c} j_{a},j_{b}\in J \end{array}}\left(w_{\tau }c^{j_{a}k}+c^{j_{a}j_{b}}\right)x_{i}^{kj_{a}j_{b}},\;\forall i\in I \end{array}$$

Equation (22) represents the deriving total cost of *i*. When $x_{i}^{kj_{a}j_{b}}$ is performed, the cost of the separated transport agent is adjusted by weight $w_{\tau }$.

## 4. Post-Auction Trajectory Planning

After generating the pre-auction trajectory, each transport agent selects a new pairing partner and node and regenerates its trajectory accordingly.

### 4.1. Auction-Based Pairing Planner

Algorithm 2 shows the overall process. After initializing the best pairing candidate set $P_{\mathrm{best}}$ to an empty state, the set of pairing candidates *P* is generated from the pre-auction trajectory $\Pi^{\mathrm{pre}}$. Each candidate comprises a transport agent separated from its original partner, a target mobile manipulator, and a pairing index indicating a node on the target manipulator’s trajectory.
**Algorithm 2** Pairing planner.  1:$P_{\mathrm{best}}\leftarrow \emptyset ,P\leftarrow GETPAIRINGCANDIDATE\left(\Pi^{\mathrm{pre}}\right)$  2:whileP≠∅do  3:  $p_{\mathrm{best}},\;J_{\mathrm{best}}^{\mathrm{done}}\leftarrow SELECTBESTPAIR\left(P,P_{\mathrm{best}},\Pi^{\mathrm{pre}},J,H\right)$  4:  $P\leftarrow EXCLUDESELECTEDAGENTS(P,p_{\mathrm{best}}.i,p_{\mathrm{best}}.\tau )$  5:  $J\leftarrow J-J_{\mathrm{best}}^{\mathrm{done}},H\leftarrow H-n\left(J_{\mathrm{best}}^{\mathrm{done}}\right)$,  6:  $P_{\mathrm{best}}\leftarrow P_{\mathrm{best}}+p_{\mathrm{best}}$  7:endwhile  8:$C^{\mathrm{init}},\;S^{\mathrm{new}}\leftarrow GETINITIALSTATEAFTERPAIRING\left(P_{\mathrm{best}}\right)$  9:$\Pi^{\mathrm{new}}\leftarrow TRJPLANNER\left(P_{\mathrm{best}},C^{\mathrm{init}},S^{\mathrm{new}},H,J\right)$10:$\Pi^{\mathrm{post}}\leftarrow REPLACETRJ\left(\Pi^{\mathrm{pre}},S^{\mathrm{new}},\Pi^{\mathrm{new}}\right)$

The algorithm iteratively selects the best available candidate until all separated transport agents in *P* are paired with new mobile manipulators. Once the best candidate $p_{\mathrm{best}}$ is identified, the robots included in $p_{\mathrm{best}}$ and the objects handled at SelectBestPair $J_{\mathrm{best}}^{\mathrm{done}}$ are excluded from further consideration. The process is then repeated with the remaining pairings to determine the next optimal pairing state.

After all pairings are finalized, the new pairing trajectory $\Pi^{\mathrm{new}}$ is generated from new starting nodes $S^{\mathrm{new}}$. By replacing the pre-auction trajectory after $S^{\mathrm{new}}$ to $\Pi^{\mathrm{new}}$, the post-auction trajectory $\Pi^{\mathrm{post}}$ is generated.

#### 4.1.1. Pairing Candidates

Algorithm 3 demonstrates the procedure for generating pairing candidates. For each transport agent τ and mobile manipulator *i*, the algorithm evaluates every node $\pi_{i}^{n}$ along *i*’s trajectory $\Pi_{i}$. If *i* is initially unpaired, the evaluation begins from the first node; otherwise, it starts from the separation index $n_{\mathrm{sep}}$.
**Algorithm 3** GetPairingCandidate  1:P←∅  2:forτ∈T,i∈Ido  3:  $P_{i,\tau }\leftarrow \emptyset$  4:  $\mathrm{for}n=0,1,...,N_{\Pi_{i}}-1\mathrm{do}$  5:    $\mathrm{if}i\in \neg I_{p}\mathrm{or}n\geq n_{\mathrm{sep}}\mathrm{then}$  6:     $t_{\tau }^{\pi_{i}^{n}}\leftarrow t_{\tau }^{\mathrm{pre}-\mathrm{sep}}+t_{\tau }^{j_{\mathrm{sep}},k_{\mathrm{sep}}}+t_{\mathrm{handle}}+t_{\tau }^{k_{\mathrm{sep}},\pi_{i}^{n}}$  7:     $\mathrm{if}t_{\tau }^{\pi_{i}^{n}}\leq t_{i}^{\pi_{i}^{n}}+t_{A}\mathrm{then}$  8:       $P_{i,\tau }\leftarrow P_{i,\tau }+\left(\tau ,i,n,t_{\tau }^{\pi_{i}^{n}}\right)$  9:     endif10:    endif11:  endfor12:  $P\leftarrow P+FILTERFASTESTMEETINGPAIRS(P_{i,\tau },N)$13:endfor14:returnP

At each node, a pairing with τ is considered feasible if the total estimated time for the transport agent to arrive—including the pre-separation time $t_{\tau }^{\mathrm{pre}-\mathrm{sep}}$, travel time from the separation point to the destination node $t_{\tau }^{j_{\mathrm{sep}},k_{\mathrm{sep}}}$, object handling time $t_{\mathrm{handle}}$, and travel time from the destination node to the *n*-th node on the trajectory $t_{\tau }^{k_{\mathrm{sep}},\pi_{i}^{n}}$—does not exceed the sum of the arrival/handling time of the target mobile manipulator at the *n*-th node $t_{i}^{\pi_{i}^{n}}$, and the allowable waiting time $t_{A}$.

Among all feasible candidates in $P_{i,\tau }$, the top *N* candidates with the earliest meeting times $t_{\tau }^{\pi_{i}^{n}}$ are selected using FILTERFASTESTMEETINGPAIRS, and the corresponding values (τ,i,n) are added to the global candidate set *P*.

#### 4.1.2. Selecting the Best Pair with Auction

To determine the optimal pairs, each candidate is evaluated using the process described in Algorithm 4.

In each evaluation, *i* in the subject candidate *p* is assumed to be paired with a transport agent at the *n*-th node on its pre-auction trajectory $\Pi_{p.i}^{\mathrm{pre}}$. Additionally, the previously selected candidates $P_{\mathrm{best}}$ are also assumed to be paired at their respective pairing nodes. Moreover, $C_{\mathrm{eval}}^{\mathrm{init}}$ and $S_{\mathrm{eval}}$ represent the initial cost and starting positions of all agents, and $J_{p}.i^{\mathrm{done}}$ represents the set of objects already handled by p.i before the *n*-th node, and they are derived using GETINITIALSTATES.

**Figure 4:**
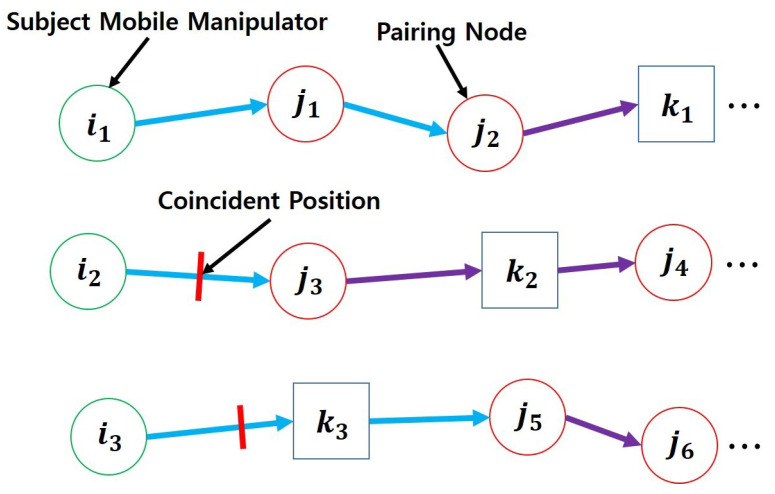
Evaluation of the subject candidate: Initial cost trajectories are depicted with blue arrows, while evaluation trajectories are represented with purple arrows.

Figure 4 shows an example of how the initial states are set when evaluating the subject candidate $p_{1}$. In this example, the mobile manipulator $i_{1}$ is paired with a transport agent at node $j_{2}$. Other relevant robots, such as $i_{2}$ and $i_{3}$, are also considered in the evaluation: $i_{2}$ is a non-subject mobile manipulator, and $i_{3}$ is a previously selected mobile manipulator that is paired with a transport agent at node $j_{5}$. When $i_{1}$ arrives at $j_{2}$, each of these robots is positioned at its respective coincident node, which is marked with a red line in the figure.
**Algorithm 4** SelectBestPair  1:$C_{\mathrm{eval}}\leftarrow \emptyset$  2:forp∈Pdo  3:  $SETPAIRINGS(p.i,p.n,P_{\mathrm{best}},\Pi_{p.i}^{\mathrm{pre}})$  4:  $C_{\mathrm{eval}}^{\mathrm{init}},S^{\mathrm{eval}},J_{p.i}^{\mathrm{done}}\leftarrow GETINITIALSTATES\left(p,P_{\mathrm{best}},\Pi^{\mathrm{pre}}\right)$  5:  $J_{\mathrm{eval}}\leftarrow J-J_{p.i}^{\mathrm{done}},\;H_{\mathrm{eval}}\leftarrow H-n\left(J_{p.i}^{\mathrm{done}}\right)$  6:  $\Pi_{p}\leftarrow TRJPLANNER\left(p,P_{\mathrm{best}},C_{\mathrm{eval}}^{\mathrm{init}},S_{\mathrm{eval}},H_{\mathrm{eval}},J_{\mathrm{eval}}\right)$  7:  $C_{\mathrm{eval}}\leftarrow C_{\mathrm{eval}}+\Pi_{p}.c$  8:endfor  9:$p_{\mathrm{best}},\;J_{\mathrm{best}}^{\mathrm{done}}\leftarrow GETMINCOSTPAIR\left(C^{\mathrm{eval}}\right)$10:$\mathrm{return}p_{\mathrm{best}},\;J_{\mathrm{best}}^{\mathrm{done}}$

In this scenario, $j_{1}$ is the object handled by the subject candidate, and $j_{2}$ becomes the starting node for $i_{1}$. For each non-subject agent, the starting node is defined as the first node on its trajectory after passing the coincident point. However, if the agent belongs to a previously selected candidate, its pairing node is used as its starting point.

Based on these updated starting nodes, the initial cost of each candidate is calculated as the sum of travel and handling times from their original starting node to the updated one, presented with blue arrows.

After defining the initial states, the candidate trajectory $\Pi_{p}$ is generated using TRJPLANNER (Section 3.2), assuming that non-subject mobile manipulators not in the previously selected set are unpaired. The resulting cost $\Pi_{p}.c$ is then computed from $\Pi_{p}$ and stored in the evaluation set $C_{\mathrm{eval}}$.

This evaluation process is repeated for all candidates. Among them, the candidate with the lowest cost is selected as the best pair $p_{\mathrm{best}}$, and its handled object set $J_{\mathrm{best}}^{\mathrm{done}}$ is excluded from consideration in the subsequent sequence auctions.

#### 4.1.3. Post-Auction Trajectory

The mobile manipulators included in $P_{\mathrm{best}}$ are designated as paired at their respective pairing nodes. Conversely, those not included in $P_{\mathrm{best}}$ are designated as unpaired, starting from their separating node if they were initially paired or from their original starting node if they were initially unpaired.

At these new starting nodes $S^{\mathrm{new}}$, the cost from each robot’s original starting node to its new starting node is set as the initial cost $C^{\mathrm{init}}$. Additionally, the object nodes between these starting nodes are omitted when planning the new trajectory.

With these initial states, the trajectory $\Pi^{\mathrm{new}}$ is generated using TRJPLANNER, and the post-auction trajectory $\Pi^{\mathrm{post}}$ is obtained by replacing the segment after $S^{\mathrm{new}}$ in $\Pi^{\mathrm{pre}}$ with $\Pi^{\mathrm{new}}$.

### 4.2. Predictor

**Figure 5:**
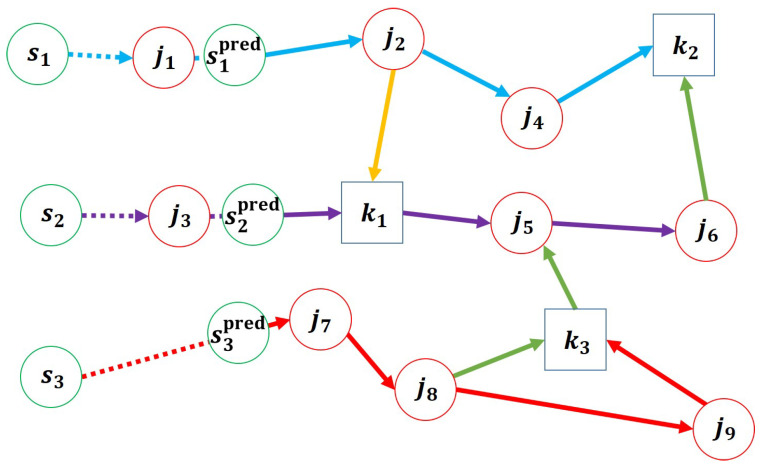
Post-auction trajectory generated from new starting nodes.

After generating the post-auction trajectory, we can create a new trajectory from the points concerning the specific time that has passed in reference to the previous starting points. As shown in Figure 5, the starting nodes move through the trajectory for the predefined prediction time, which is indicated with a dotted arrow, and the predictive starting node is set as $s_{i}^{\mathrm{pred}}$. From these nodes, a new trajectory is generated.

As performed in Section 4.1.2, each mobile manipulator designates its last accessing node during the prediction time as its predictive initial accessing nodes, and the sum of the traveling time and handling time from each predictive starting node to the initial accessing node is defined as the initial cost $c_{i}^{\mathrm{init}}$. For instance, in Figure 5, $j_{2}$, $k_{1}$, and $j_{7}$ are assigned as the new starting nodes for $i_{1}$, $i_{2}$, and $i_{3}$, respectively, and the costs from each $s_{i}^{\mathrm{pred}}$ to its corresponding starting node are set as $c_{i}^{\mathrm{init}}$.

If the initial accessing node for *i* is an object node *j*, then $x_{i}^{sj}$ is set to 1. In addition, object nodes handled by each paired robot within the prediction range are added to its initially loaded objects $l_{i}^{\mathrm{init}}$ in the new trajectory. As shown in Figure 5, $j_{1}$ and $j_{3}$ are added to the loaded objects of $i_{1}$ and $i_{2}$, respectively, and $x_{i_{1}}^{sj_{2}}$ and $x_{i_{3}}^{sj_{7}}$ are set to 1.

## 5. Experiments

The experiments were conducted using Webots [25], an open-source robotic simulator integrated with ROS2 [26].

To generate navigation trajectories, we employed the A* [27] algorithm to compute travel paths and estimate the costs between waypoints, including object positions, destinations, and robot locations. Based on the computed costs, the Gurobi Optimizer [28] was used to generate optimized trajectories as described in Section 3.2, where the weight parameter *w* was set to 0.7. In addition, ORCA (Optimal Reciprocal Collision Avoidance) [29] was applied during navigation to prevent collisions between robots.

The experiments are designed to evaluate the proposed system from two perspectives—its scalability in different task settings, and the impact of the auction-based pairing algorithm on overall performance.

**Figure 6:**
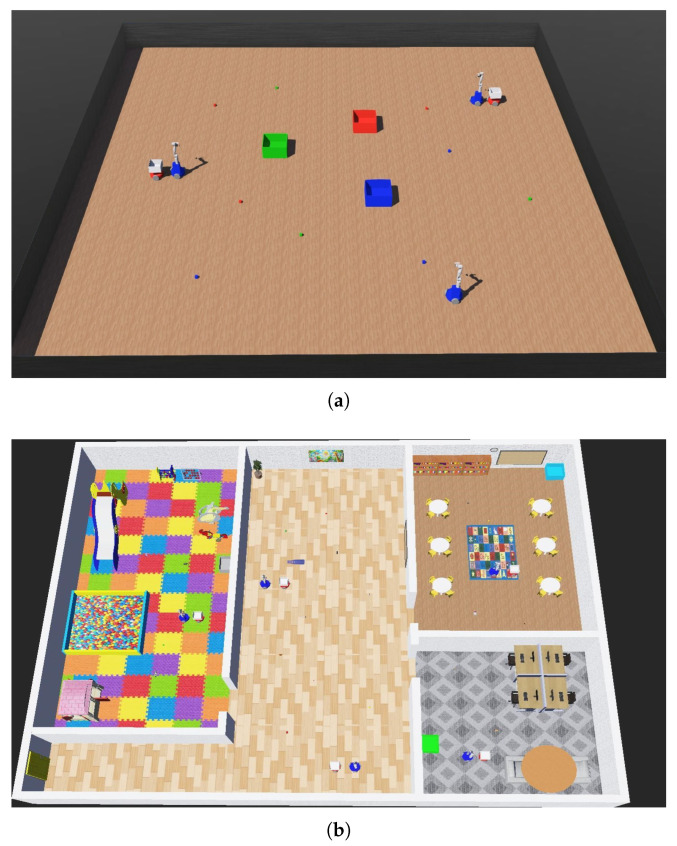
Simulation environment used in the experiments: (**a**) simplified environment; (**b**) kindergarten-themed environment.

In evaluating its scalability, the experiments were conducted in two environments: a simplified environment and a kindergarten-themed environment—both shown in Figure 6. The simplified environment consists of cube-shaped objects and three destinations without obstacles. In contrast, the kindergarten-themed environment includes objects commonly found in kindergartens, four destinations, and various obstacles.

Meanwhile, to evaluate the effectiveness of the auction-based pairing algorithm, we compared the proposed system to a baseline configuration that does not include this mechanism. In the baseline system, each mobile manipulator operates independently with fixed storage, and no dynamic role reassignment with transport agents occurs.

Both experiments were conducted under varying numbers of objects $N_{j}$ and transport agents $N_{\tau }$, assuming $L^{\max}=2$ for all transport agents.

For a visual representation of the experiments, all corresponding video recordings can be accessed at the following link: (https://youtube.com/playlist?list=PLB1pUAsYGpRHK9mhHIeu827JLSoLhcznD&si=97sXqhfHEqkMkMc0, accessed on 20 May 2025).

### 5.1. Scalability Evaluation

#### 5.1.1. Experimental Scenarios

To evaluate the scalability of the proposed system, we test its performance under two different environments:

- **Simplified Environment**: A minimal environment shown in Figure 6a, where three mobile manipulators and a varying number of transport agents collaborate to tidy up the workspace by placing cube-shaped objects into the destination zones of matching colors.
- **Kindergarten-Themed Environment**: A realistic environment shown in Figure 6b, which consists of four distinct areas: a playroom, a corridor, a classroom, and a teacher’s room. In this environment, five mobile manipulators and a varying number of transport agents work together to tidy up four different types of objects—balls, toys, teaching aids, and stationery—by delivering them to their designated locations. To ensure consistent handling and loading behaviors, all objects were standardized to have a weight of 50 g and were encapsulated in a cubic bounding box with a side length of 0.07 m.

#### 5.1.2. Performance Comparison

**Figure 7:**
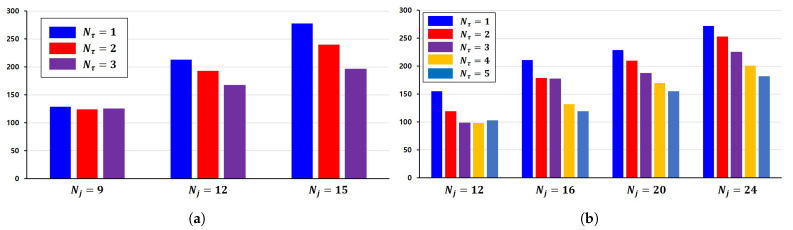
Changes in task completion time depend on the number of transport agents and objects: (**a**) simplified environment; (**b**) kindergarten-themed environment.

We measured task completion time in both environments while increasing $N_{j}$ and $N_{\tau }$. The results, summarized in Figure 7 and Table 2, show that the proposed system scales well in both structured and complex environments.

In the simplified environment, the system shows relatively minor gains when $N_{j}$ is small. For example, at $N_{j}=9$, increasing $N_{\tau }$ from 2 to 3 results in only a 1-s increase, suggesting possible coordination overhead. However, as the number of objects increases to $N_{j}=15$, the benefit becomes more evident, with up to 44 s of reduction when increasing $N_{\tau }$ from 2 to 3.

In the kindergarten-themed environment, which introduces additional complexity, such as obstacles and spatial constraints, similar trends are observed. For $N_{j}=12$, increasing the number of transport agents from one to two reduces the completion time significantly by 36 s. A further increase to three units continues to reduce the time to 98 s. However, increasing $N_{\tau }$ from 3 to 4 does not yield any improvement, and adding a fifth transport agent increases the completion time by 5 s, possibly due to the coordination overhead in a low-demand scenario.

For larger workloads, $N_{j}=20$ and $N_{j}=24$, the performance improves more consistently as the number of transport agents increases, following a similar trend observed in the simplified environment. For $N_{j}=20$, task completion time drops from 229 s with one unit to 155 s with five units, with the average decrease being 18.5 s per unit. A similar pattern is observed at $N_{j}=24$, where time reduces from 272 to 182 s as the number of transport agents increases, with the average decrease being 22.5 s per unit.

These results demonstrate that the proposed system scales effectively in both simplified and realistic settings, and that the degree of performance improvement becomes more substantial as the task complexity and workload increase.

### 5.2. Effectiveness of Auction-Based Pairing

#### 5.2.1. Experimental Scenarios

To evaluate the effectiveness of the auction-based pairing algorithm, we define two experimental scenarios:

- **With auction-based pairing**: Robots use the proposed dynamic pairing strategy, where transport agents and mobile manipulators are paired through an auction-based algorithm. Pairings can change dynamically based on task status and robot availability.
- **Without auction-based pairing**: Robots operate without the auction-based pairing mechanism. Instead of dynamic pairing, each mobile manipulator, assumed to be paired with a transport agent, is equipped with a container that has the same $L^{\max}$ as a transport agent and performs the task without any pairing mechanism. The trajectory planner employed in this work is based on the approach proposed in [14].

Both scenarios are tested under identical environment settings, including object placements and robot configurations. Task completion time is measured and compared to analyze the effectiveness of the auction-based pairing strategy.

#### 5.2.2. Performance Comparison

To assess the effectiveness of the proposed auction-based pairing strategy, we compare its performance against a baseline configuration in which each mobile manipulator is equipped with a fixed container and no dynamic pairing is allowed. Both systems are evaluated in the kindergarten-themed environment.

**Figure 8:**
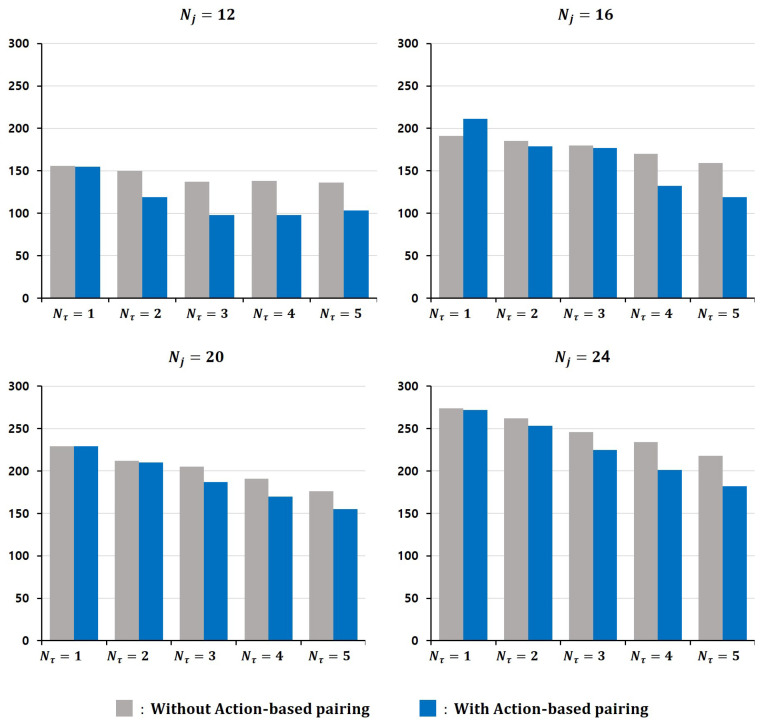
Visual comparison of task completion times according to the number of transport agents and objects.

As shown in Figure 8 and Table 3, the auction-based system achieves better performance in most settings, particularly when the number of transport agents increases.

For example, at $N_{j}=12$, the completion time decreases from 156 s to 103 s as $N_{\tau }$ increases from 1 to 5 without an auction-based pairing; with the proposed method, the time drops from 155 s to 98 s over the same range.

At $N_{j}=16$, the difference becomes even more pronounced. The baseline shows a reduction of 32 s (from 191 s to 159 s), while the auction-based method achieves a 92 s reduction (from 211 s to 119 s), demonstrating more effective resource utilization under moderate task loads.

Even in higher workloads, the auction-based method consistently maintains a more significant margin of improvement. When $N_{j}=24$, the completion time drops from 274 s to 218 s without an auction-based pairing and from 272 s to 182 s with an auction-based pairing, yielding a 36 s advantage.

These results indicate that the proposed auction-based pairing enables better adaptation to varying task distributions. By allowing dynamic reallocation of roles between mobile manipulators and transport agents, the system achieves consistently shorter completion times across different conditions.

## 6. Discussion

This study proposes an MAPD system that enables efficient collaboration between mobile manipulators and transport agents. Unlike conventional approaches that treat heterogeneous agents separately, our system integrates trajectory optimization with auction-based dynamic pairing to support flexible role adjustment and synergy.

Experimental results validate the approach, showing that the proposed method improves task completion time, especially in high-demand situations. These improvements highlight the value of strategic dynamic pairing and partial trajectory planning in optimizing heterogeneous multi-robot collaboration.

However, the current system assumes that all transport agents have identical capabilities and ignores energy limitations. These assumptions simplify the problem but may limit applicability in real-world deployments. Future improvements will focus on addressing these constraints by incorporating robot heterogeneity and energy-aware task allocation.

## 7. Conclusions

This work presented an MAPD framework that enables heterogeneous multi-robot collaboration through dynamic pairing and partial trajectory planning. By integrating an auction-based planner with trajectory optimization, the system achieves improved task efficiency and flexibility. Experimental validation in both simplified and realistic environments confirmed the effectiveness of the proposed method under various workloads.

For future work, we plan to extend the system to consider energy-aware scheduling [30,31,32], which is essential for real-world robot operation, by incorporating battery levels and standby agents in charging stations, allowing for dynamic deployment based on resource availability and demand. Furthermore, the current system assumes uniform robot capabilities. Our future research will explore heterogeneous agents with varying speeds and capacities to enhance generalizability.

## Figures and Tables

**Table 1:** Definitions of the notations.

Parameters	
$I=\{i_{1},i_{2},..,i_{n}\}$	Set of mobile manipulator *i*.
$I_{p}\subseteq I$	Set of mobile manipulators paired with transport agent.
$J=\{j_{1},j_{2},..,j_{m}\}$	Set of object node *j*.
w∈[0,1]	Completion time weight.
$w_{\tau }\in \left[0,1\right]$	Separated transport agent cost weight.
*k*	Destination node.
H∈N	Object number horizon.
**Constraints**	
$l_{i}^{\mathrm{init}}\in Z_{\geq 0}$	Initial loaded object of the paired transport agent of the mobile manipulator *i*.
$L^{\max}\in N$	Maximum carrying capacity of a transport agent.
$c_{i}^{\mathrm{init}}\in R_{\geq 0}$	Initial cost of the mobile manipulator *i*.
$c_{i}^{sj}\in R_{\geq 0}$	The cost when traveling $\left(s_{i},j\right)$ and handling *j*.
$c^{jk}\in R_{\geq 0}$	The cost when traveling (j,k) and placing the object *j* at its destination node *k*.
$c^{kj}\in R_{\geq 0}$	The cost when traveling (k,j) and handling *j*.
$c^{jj^{\prime }}\in R_{\geq 0}$	The cost when traveling $\left(j,j^{\prime }\right)$ and handling $j^{\prime }$.
**Decision variables**	
$x_{i}^{sj}\in \left\{0,1\right\}$	1 if *i* travels $\left(s_{i},j\right)$ and picks up *j*, 0 otherwise.
$x_{i}^{jkj^{\prime }}\in \left\{0,1\right\}$	1 if *i* travels (j,k), places the *j* at its destination node *k*, travels $\left(k,j^{\prime }\right)$, and picks up $j^{\prime }$, 0 otherwise.
$x_{i}^{jj^{\prime }}\in \left\{0,1\right\}$	1 if *i* travels $\left(j,j^{\prime }\right)$ and picks up $j^{\prime }$, 0 otherwise.
$x_{i}^{kjj^{\prime }}\in \left\{0,1\right\}$	1 if *i* separates its paired transport and the transport agent travels to *k*, the destination node of *j*, and travels $\left(j,j^{\prime }\right)$ and picks up $j^{\prime }$, 0 otherwise.
$x_{i}^{jk}\in \left\{0,1\right\}$	1 if *i* travels (j,k) at last and places the *j* at its destination node *k*, 0 otherwise.
$o_{i}^{j}\in R_{\geq 0}$	The order of picking up *j* among the tasks of *i*.
$C_{i}\in R_{\geq 0}$	The total cost of *i*.

**Table 2:** Detailed task completion times by the number of transport agents and objects.

$N_{\tau }$	Simplified Environment			Kindergarten-Themed Environment			
	$N_{j}=9$	$N_{j}=12$	$N_{j}=15$	$N_{j}=12$	$N_{j}=16$	$N_{j}=20$	$N_{j}=24$
1	129	213 s	278 s	155 s	211 s	229 s	272 s
2	124 s	193 s	240 s	119 s	179 s	210 s	253 s
3	125 s	167 s	196 s	98 s	177 s	187 s	225 s
4	–			98 s	132 s	170 s	201 s
5	–			103 s	119 s	155 s	182 s

**Table 3:** Detailed task completion times by the number of transport agents and objects.

$N_{\tau }$	Without Auction-Based Pairing				With Auction-Based Pairing			
	$N_{j}=12$	$N_{j}=16$	$N_{j}=20$	$N_{j}=24$	$N_{j}=12$	$N_{j}=16$	$N_{j}=20$	$N_{j}=24$
1	156 s	191 s	229 s	274 s	155 s	211 s	229 s	272 s
2	150 s	185 s	212 s	262 s	119 s	179 s	210 s	253 s
3	137 s	180 s	205 s	246 s	98 s	177 s	187 s	225 s
4	138 s	170 s	191 s	234 s	98 s	132 s	170 s	201 s
5	136 s	159 s	176 s	218 s	103 s	119 s	155 s	182 s

## Data Availability

Data is contained within the article.

## Abbreviations

The following abbreviations are used in this manuscript:

MAPD	Multi-Agent Pickup and Delivery
MTZ	Miller–Tucker–Zemlin
ROS	Robot operating system
A*	A-star
ORCA	Optimal Reciprocal Collision Avoidance
